# Factors associated with headache and neck pain among telecommuters – a five days follow-up

**DOI:** 10.1186/s12889-021-11144-6

**Published:** 2021-06-06

**Authors:** Mariève Houle, Arianne Lessard, Émile Marineau-Bélanger, Arnaud Lardon, Andrée-Anne Marchand, Martin Descarreaux, Jacques Abboud

**Affiliations:** 1 Department of Anatomy, Université du Québec à Trois-Rivières, 3351, boul. des Forges, C.P. 500, Trois-Rivières, QC G8Z 4M3 Canada; 2 Department of Human Kinetics, Université du Québec à Trois-Rivières, 3351, boul. des Forges, C.P. 500, Trois-Rivières, QC G8Z 4M3 Canada; 3 Institut Franco-Européen de Chiropraxie, 24, Blvd Paul Vaillant-Couturier, 94200, Ivry sur Seine, France; 4 Department of Chiropractic, Université du Québec à Trois-Rivières, 3351, boul. des Forges, C.P. 500, Trois-Rivières, QC G8Z 4M3 Canada

**Keywords:** Telecommuting, Physical health, Disability, Headset, Headache, Neck pain, COVID-19

## Abstract

**Background:**

The current sanitary crisis brought on by the COVID-19 recently forced a large proportion of workers to adopt telecommuting with limited time to plan transition. Given that several work-related risk factors are associated with headache and neck pain, it seems important to determine those associated with headache and neck pain in telecommuters.

The main objective of this study was to identify which telecommuting and individual associated factors are related with headache and neck pain occurrence in telecommuters over a five days follow-up. The second objective was to evaluate the impact of wearing a headset on headache and neck pain intensity in telecommuters.

**Methods:**

One hundred and sixty-two participants in telecommuting situation were recruited. Baseline assessment included sociodemographic data, headache and neck pain-related disability (6-item Headache Impact Test (HIT-6) and Neck Bournemouth Questionnaire (NBQ)), headache and neck pain frequency and intensity as well as questions about the wearing of a headset (headset wearing, headset type and headset wearing hours). A prospective data collection of headache, neck pain and headset wearing was conducted using daily e-mail over a 5-day follow-up. A stepwise multivariate regression model was performed to determine associated factors of headache or neck pain occurrence during the follow-up. A t-test was conducted to assess the impact of headset wearing on headache and neck pain intensity during the follow-up.

**Results:**

Regarding headache, the stepwise multivariate regression model showed that the HIT-6 score was associated with future headache occurrence in telecommuters (OR (95% CI) = 1.094 (1.042–1.148); *R*^*2*^ = 0.094; *p* <  0.001). For neck pain, the stepwise multivariate regression showed that the NBQ score was related to future neck pain occurrence in telecommuters (OR (95% CI) = 1.182 (1.102–1.269); *R*^*2*^ = 0.182; *p* <  0.001). T-test showed no difference between participants that wore a headset and participant that did not wore a headset on mean headache (*p* = 0.94) and neck pain (*p* = 0.56) intensity during the five days follow-up.

**Conclusion:**

Although several work-related risk factors are associated with headache and neck pain in workers, telecommuting did not present the same risks. Working set-up did not have a significant impact on headache and neck pain as headache-related disability was the only associated factor of future headache episodes and neck-pain related disability was the only associated factor of future neck pain episodes. Also, wearing a headset had no impact on headache and neck pain in telecommuters.

## Introduction

Information and communication technologies (ICT) have evolved greatly over the past 10 years and many employers now support or promote their employees to work from home. According to a narrative review published in 2015, telecommuting sometimes referred to as telework can be defined as a work practice that involves members of an organization substituting a portion of their typical work hours to work away from a central workplace—principally from home—using technology to interact with others as needed to conduct work tasks [1]. Although the prevalence of telecommuting remains difficult to establish, it was estimated that nearly 60% of U.S. organizations allowed their employees to do some form of telecommuting in 2014 [1]. In addition to reducing company costs related to space rental, telecommuting appears to increase workers’ well-being, autonomy and productivity, improve work-family balance and reduce work-related stress [2, 3].

Even though telecommuting is becoming a new reality for many employees around the globe, several issues with regards to work-related health have been raised. A recent rapid review investigated the impact of telecommuting on individual workers’ mental and physical health [4]. Twenty-three studies were included in the review and several health-related outcomes were looked at including pain, self-reported health and well-being. Among the reported results, the authors found that only two studies investigated pain outcomes. One study reported lower pain levels in male telecommuters compared to commuters [5] while the other study found no difference between commuters and telecommuters [6]. Evidence regarding physical outcomes and telecommuting was scarce and surprising to the authors given that physical health is a broad and well-studied topic in work ergonomics.

Musculoskeletal pain often affects workers and it is estimated that more than 30% of the workforce report pain several times a week [7]. Primary headaches and neck pain are among the most common work-related health conditions [8, 9] and both are highly prevalent in workers. Neck pain and primary headaches one-year prevalence is respectively estimated at around 48 and 46% in the worker population [10, 11]. Current evidences suggest that risk factors for neck pain and headache carry both non-modifiable or modifiable risk factors. For both conditions, sex, age, previous musculoskeletal symptoms and history of head or neck injury are all individual risk factors of developing headache or neck pain [10, 12–14]. In workers, and more specifically office workers, prolonged work position, repetitive movements, posture (neck and head), workstation design (keyboard position, mouse position, screen height) have all been associated with neck pain [10, 12]. Long working hours in the same position including computer work is considered a headache risk factor [11].

Given that several work-related risk factors are associated with headache and neck pain, it seems important to identify those of headache and neck pain in a telecommuting context including associated factors related to ergonomics and working conditions. This is particularly relevant given that the current sanitary crisis suddenly catapulted a significant proportion of workers in full-time telecommuting with very limited or no time to plan the transition. Although working from home was proposed to be a very effective tool for reducing the infection rate [15], several telecommuters were forced to work in improvised home offices (kitchen, living room, office at home) for long hours with limited access to proper furniture and proper ICT equipment and resources. According to Lopez-Leon and colleagues, recommendation to work from home during the COVID-19 pandemic included establishing a dedicated workspace with comfortable chair, proper lighting and ventilation and adequate accessories such as a microphone, camera and noise-cancelling headphones [16]. Many telecommuters now use computer headsets during their online meeting and overall telecommuting activities. The use of headsets creates an additional mechanical stress by adding weight on the head that in turn must be supported by neck muscles and passive structures. Using a wearable device such as a headset combined with an inadequate posture while working will force the wearer to adopt adaptative postures that can provoke localized muscle fatigue [17] leading to a potential headache or neck pain episode.

The main objective of this study was to identify which telecommuting and individual factors are associated with headache and neck pain occurrence in telecommuters during a 5-day follow-up. A secondary objective of the study was to evaluate the impact of wearing a headset on headache and neck pain intensity in telecommuters. We hypothesized that previous experience of headache or neck pain combined with long working hours and an inappropriate working set-up will be related to headache and neck pain in telecommuters. We also hypothesized that wearing a headset will have an impact on headache and neck pain intensity in telecommuters.

## Methods

### Study design

This study is a prospective observational cohort study. Recruitment, data collection and follow-up were conducted online from May 2020 to July 2020.

### Participants

One hundred and sixty-two participants were recruited via social media platforms (Facebook pages and University web platforms). To be included in the study, participants had to be aged between 18 and 65 years old and in a full-time telecommuting situation since at least one week prior to enrollment. Participants were not eligible if they had a head and/or neck trauma in the past 6 months. The project received approval from the Human Research Ethics Board of “Université du Québec à Trois-Rivières” (CER-20-266-07.10) and all methods were performed in accordance with the relevant guidelines and regulations. All participants provided informed written consent before completing the baseline assessment.

### Data collection

#### Baseline assessment and clinical outcomes

Participants were asked to first fill in  an online survey (SurveyMonkey Inc., San Mateo, California, USA) as a baseline data collection which included sociodemographic information such as height, weight, age and gender. Participants were then asked about their workstation including questions about the presence of one designated place at home and if they wore a headset during the 7 days prior to enrollment and the headset type. In-ear and over-ear headset types were differentiated to assess the weight variation of headset on headache and neck pain occurrence. Participants also completed a brief clinical history in order to assess headache and neck pain frequency and mean pain intensity over the 7 days prior to enrollment as well as headache and neck pain-related disability.

Headache was defined as the presence of pain or discomfort in any region of the head [18] without distinction to the underlying diagnoses. Neck pain was defined as the presence of pain or discomfort localised from the superior nuchal line and external occipital protuberance inferiorly to the spine of the scapula, along the superior border of the clavicle to the suprasternal notch [19]. To avoid confusion between these two sites, an illustration of the head and the neck with boundaries of each region clearly delineated was added to the survey.

Headache and neck pain episodes were assessed using the following items: [A] Headache and neck pain intensity was measured using a numerical rating scale (NRS), anchored between 0 (no pain) and 10 (worst possible pain). [B] The validated French version of the 6-item Headache Impact Test (HIT-6) questionnaire [20] was used to provide a global measure of adverse headache impact [21]. The total score is calculated by adding scores from each of the 6 items (pain, social functioning, role functioning, vitality, cognitive functioning and psychological distress) for a maximum score of 78 points where greater score indicates greater adverse impact. Furthermore, the total score allows for a division of the headache-related adverse impact into four categories: headaches having a major impact (score ≥ 60), important impact (score = 56 to 59), some impact (score = 50 to 55) and weak impact (score ≤ 49) on daily life. [C] The validated French version of the Neck Bournemouth Questionnaire (NBQ) was also used to document neck pain and function [22]. The NBQ consists of seven questions, each one focusing on a different pain dimension (pain intensity, functional status in daily living and social activities, affective dimensions of anxiety and depression, cognitive aspects of fear-avoidance belief and pain locus of control). Each question is rated on a NRS with possible answers ranging from 0 (much better) to 10 (much worsen). The total NBQ score is calculated by summing the score of all seven questions for a maximum score of 70 with higher score indicating greater impact of neck pain on participants’ lives.

#### 5-day follow-up

**Fig. 1:**
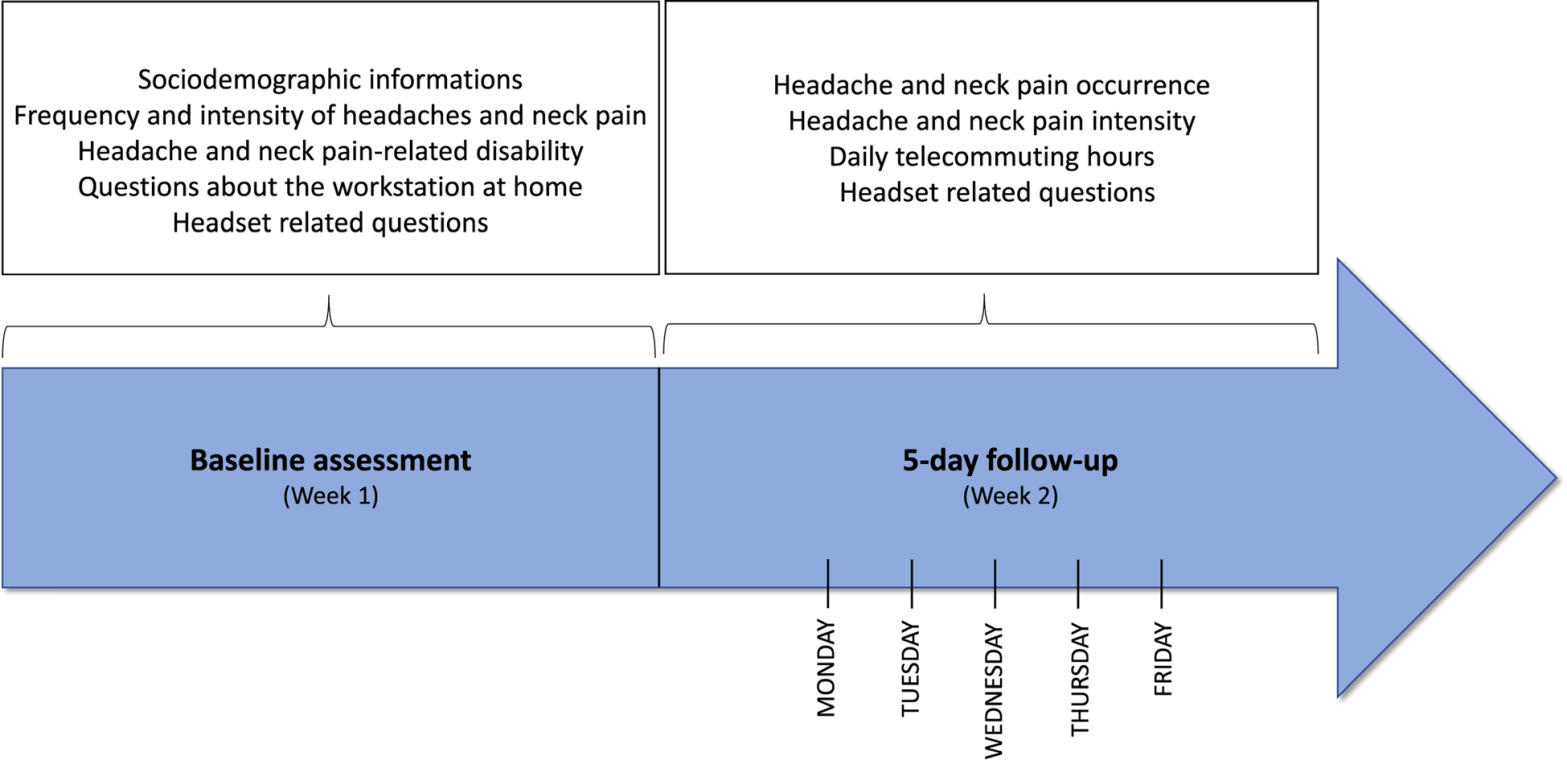
Timeline of data collection

A daily online survey (SurveyMonkey Inc., San Mateo, California, USA) was sent to all participants who completed the baseline data collection. Participants received a daily email on 5 consecutive working days at 5 p.m., starting on the next Monday following the completion of the baseline data collection. Participants were asked about the number of daily telecommuting hours (including bouts of interrupted work time), having wore a headset during telecommuting and the number of headset wearing hours during telecommuting. Participants were also asked about the occurrence of headache or neck pain during the day. If so, participants were invited to report the headache or the neck pain intensity using a NRS as described previously and if they took any medication to reduce their pain. Figure 1 illustrates the complete data collection timeline.

### Independent and dependent variables

All independent and dependent variables are presented in Table 1. Regarding independent variables, height, age, gender, baseline headache and neck pain-related disability, baseline headache and neck pain frequency, and baseline headache and neck pain intensity were considered as non-modifiable factors. Weight, BMI, home workstation, baseline headset wearing, headset type as well as follow-up headset wearing, follow-up telecommuting hours, and follow-up headset wearing hours were considered as modifiable factors.

**Table 1:** Independent and dependent variables definitions

**Objective 1. Determine which telecommuting and individual factors associated with headache and neck pain occurrence in telecommuters.**		
**Independent variables**	**Definitions**	**Time of data collection**
Height	Height (m)	*Baseline*
Weight	Weight (kg)	
BMI	BMI (kg/m^2^)	
Age	Age (years)	
Gender	M: F: O	
HIT-6	Headache-related disability	
NBQ	Neck pain-related disability	
Baseline headache frequency	Number of headache episodes during the 7 days prior to enrollment	
Baseline headache intensity	Mean headache intensity during the 7 days prior to enrollment	
Baseline neck pain frequency	Number of headache episodes during the 7 days prior to enrollment	
Baseline neck pain intensity	Mean neck pain intensity during the 7 days prior to enrollment	
Home workstation	Presence of one designated place at home where the participant was telecommuting	
Baseline headset wearing	Having wore a headset during the 7 days prior to enrollment *(yes or no question)*	
Headset type	In-ear or over-ear headset	
Follow-up headset wearing	Having wore a headset at least one time during the follow-up *(yes or no question)*	*Follow-up*
Follow-up telecommuting hours	Mean telecommuting hours based on daily survey	
Follow-up headset wearing hours	Mean headset wearing hours based on daily survey	
**Dependent variables**	**Definitions**	
Follow-up headache occurrence	Having at least one headache episode during the follow-up *(yes or no question)*	*Follow-up*
Follow-up neck pain occurrence	Having at least one neck pain episode during the follow-up *(yes or no question)*	
**Objective 2. Evaluate the impact of wearing a headset on headache and neck pain intensity in telecommuters.**		
**Independent variable**	**Definition**	**Time of data collection**
Follow-up headset wearing	Having wore a headset at least one time during the follow-up *(yes or no question)*	*Follow-up*
**Dependent variables**	**Definitions**	
Follow-up headache intensity	Mean headache intensity based on the number of headache episodes during the follow-up	*Follow-up*
Follow-up neck pain intensity	Mean neck pain intensity based on the number of neck pain episodes during the follow-up	

BMI = Body Mass Index, M = Male, F = female, O = other gender (not identifying as either male of female), HIT-6 = 6-Item Headache Impact Test, NBQ = Neck Bournemouth Questionnaire

### Statistical analysis

#### Baseline assessment and clinical variables

Normality of the distribution of each variable was assessed with the Shapiro-Wilk test and by visual inspection of corresponding histograms. Means and standard deviations were calculated for continuous baseline variables (height, weight, BMI, age, HIT-6 and NBQ scores and baseline headache and neck pain intensity) while proportions were reported for dichotomous baseline variables (baseline headache and neck pain frequency, home workstation and baseline headset wearing) for all participants. Responders (participants that completed the 5-day follow-up) and non-responders (participants that did not complete the 5-day follow-up) were compared for baseline characteristics using t-tests. 

#### Associated factors of headache occurrence during the 5-day follow-up

To assess associated factors of headache occurrence, only data from participants that completed the follow-up were considered. We first divided participants into two groups (participants reporting no headache episode and participants reporting at least one headache episode during the 5-day follow-up). Bivariate analyses were performed to determine the association between the potential related factors (modifiable or non-modifiable) and the presence of headache. Chi-square tests were used for dichotomous variables while t-tests (parametric variables) and Mann-Withney Wilcoxon tests (non-parametric variables) were used for continuous variables. An independent variable was considered a potential associated factor of headache occurrence and included in the binary logistic regression for the stepwise multivariate model only if the degree of statistical significance was equal or inferior to 0.2. Clinical variables including HIT-6, NBQ, baseline headache and neck pain intensity and frequency were then screened for collinearity using Spearman rank test. A correlation coefficient value superior to 0.40 indicates that the variable needed to be removed from the model. Then, one by one, all non-significant associated factors (*p* > 0.05) were removed from the model starting with the one with the lowest association level to end up with a model comprising only significant associated factors. Odds Ratios (95% CI) were indicated to report the strength of this association.

#### Associated factors of neck pain occurrence during the 5-day follow-up

To assess associated factors of neck pain occurrence, only data from participants that completed the follow-up were considered. Participants were first divided into two groups (participants reporting no neck pain and participants reporting at least one neck pain episode during the follow-up). Statistical analyses identical to the ones previously decribed were conducted to assess neck pain associated factors.

To answer our second objective, t-tests for independent groups were conducted to compare headache or neck pain mean intensity between participants wearing a headset and participants not wearing a headset during the follow-up. Analyses were performed using STATISTICA statistical package version 10 (Statsoft, Tulsa, OK) and STATA.12® (StataCorp, Texas, USA). The level of significance was set at *p*-value ≤ 0.05 except for the bivariate correlation tests as previoulsy described.

## Results

### Baseline demographics

One hundred and sixty-two participants (49 males, 112 females and 1 not identifying as either male or female) were recruited for the study and included in the data analyses (Table 2).

**Table 2:** Baseline demographic data for responders and non-responders

	Responders (***n*** = 109) (mean ± sd)	Non-responders (***n*** = 53) (mean ± sd)
**Gender (M: F: O)**	39: 69: 1	10: 43: 0
**Height (m)**	1.69 ± 0.10	1.74 ± 0.50
**Weight (kg)**	71.16 ± 17.03	69.21 ± 17.85
**BMI (kg/m**^**2**^**)**	24.85 ± 4.71	24.27 ± 6.55
**Age (years)**	36.24 ± 11.05	35.21 ± 10.26
**HIT-6 (/78)**	49.39 ± 9.37	50.96 ± 9.97
**NBQ (/70)**	15.32 ± 12.23	21.15 ± 12.95
**Baseline headache intensity**	2.40 ± 2.25	2.79 ± 2.48
**Baseline neck pain intensity**	2.30 ± 2.09	3.40 ± 2.48
**Baseline headache occurrence**		
*Yes (%) / No (%)*	66.97 / 33.03	69.81 / 30.19
**Baseline neck pain occurrence**		
*Yes (%) / No (%)*	65.14 / 34.86	74.47 / 24.53
**Home workstation**		
*Yes (%) / No (%)*	28.44/ 71.56	33.96/ 66.04
**Baseline headset wearing**		
*Yes (%) / No (%)*	73.30 / 26.70	74.47 / 24.53

M = male, F = female, O = other gender (not identifying as either male of female), BMI = body mass index, HIT-6 = 6-item headache impact test, NBQ = Neck Bournemouth Questionnaire, sd = standard deviation.

T-test results for independent variables showed no significant differences between participants that completed the follow-up and non-responders for height, weight, BMI, age, HIT-6 and baseline headache intensity (all *p*-values > 0.311). However, NBQ was higher in non-responders (*p* = 0.006) as well as baseline neck pain intensity (*p* = 0.004).

###  Associated factors of headache during the 5-day follow-up

Out of the 109 participants, 67 reported at least one headache episode during the follow-up compared to 42 who reported no headache episode. Demographic data for each group and bivariate associations for all independent variables are presented in Table 3.

**Table 3:** Demographic data for participants without headache and participants with follow-up headache occurrence

	Without headache during the follow-up (***n*** = 42) (mean ± sd)	With at least one headache episode during the follow-up (***n*** = 67) (mean ± sd)	Chi-square (***p***-value)	T-test or Wilcoxon (*p*-value)
*Baseline variables*				
**Gender (M: F: O)**	19: 22: 1	20: 47: 0	0.101	–
**Height (m)**	1.70 ± 0.11	1.67 ± 0.09	–	0.073^†^
**Weight (kg)**	74.34 ± 15.50	69.17 ± 17.72	–	0.030^†^
**BMI (kg/m**^**2**^**)**	25.38 ± 4.71	24.52 ± 4.71	–	0.215^†^
**Age (years)**	37.83 ± 11.03	35.24 ± 11.03	–	0.153^†^
**HIT-6 (/78)**	45.07 ± 8.83	52.10 ± 8.70	–	< 0.001^†^
**NBQ (/70)**	12.00 ± 12.02	17.40 ± 11.97	–	0.011^†^
**Baseline headache intensity**	1.00 ± 1.50	3.28 ± 2.20	–	< 0.001^†^
**Baseline neck pain intensity**	1.50 ± 1.95	2.81 ± 2.03	–	0.004^†^
**Baseline headache frequency**	0.83 ± 1.48	2.10 ± 1.74	–	< 0.001^†^
**Baseline neck pain frequency**	1.62 ± 2.26	2.94 ± 2.58	–	0.001^†^
**Home workstation** *Yes (%) / No (%)*	64.29 / 35.71	76.12 / 23.88	0.183	–
**Baseline headset wearing** *Yes (%) / No (%)*	52.38 / 47.62	70.15 / 29.85	0.061	–
*Follow-up variables*				
**Follow-up headset wearing** *Yes (%) / No (%)*	54.76 / 45.24	62.69 / 37.31	0.412	–
**Follow-up telecommuting hours**	5.13 ± 2.04	5.50 ± 1.91	–	0.339*
**Follow-up headset wearing hours**	0.85 ± 1.49	1.70 ± 2.35	–	0.105^†^

M = male, F = female, O = other gender (not identifying as either male of female), BMI = body mass index, HIT-6 = 6-item headache impact test, NBQ = Neck Bournemouth Questionnaire, sd = standard deviation. * = t-test, ^†^ = Wilcoxon test

Following the Spearman rank test analysis, only the HIT-6 was retained to represent the clinical baseline variables related to headache occurrence. The final stepwise multivariate regression model to determine factors related to headache occurrence and the corresponding odds ratio are described in Table 4. At the end, 9.4% of the variance could be explained by the final model.

**Table 4:** Odds ratios, confidence intervals and *p*-values of factors associated with headache occurrence retained in the final stepwise model

Variable	Odds Ratio (95% CI)	P-value	Multivariate regression
**HIT-6**	1.094 (1.042–1.148)	< 0.001	R^2^ = 0.094

HIT-6 = 6-item headache impact test, CI = Confidence Interval

### Associated factors of neck pain during the 5-day follow-up

Out of the 109 responders, 77 reported at least one neck pain episode during the follow-up compared to 32 who reported no neck pain episode. Demographic data and bivariate associations for each group are presented in Table 5.

**Table 5:** Demographic data for participants without neck pain and participants with neck pain occurrence during the 5-day follow-up

	Without neck pain during the follow-up (***n*** = 32) (mean ± sd)	With at least one neck pain episode during the follow-up (***n*** = 77) (mean ± sd)	Chi-square (***p***-value)	T-test or Wilcoxon (*p*-value)
*Baseline variables*				
**Gender (M: F: O)**	14: 18: 0	25: 51: 1	0.455	–
**Height (m)**	1.69 ± 0.09	1.68 ± 0.09	–	0.622^†^
**Weight (kg)**	71.92 ± 12.41	70.86 ± 16.43	–	0.316^†^
**BMI (kg/m**^**2**^**)**	25.10 ± 3.45	24.91 ± 4.66	–	0.220^†^
**Age (years)**	41.06 ± 12.49	35.56 ± 10.99	–	0.005^†^
**HIT-6 (/78)**	45.19 ± 8.26	49.30 ± 9.56	–	0.003^†^
**NBQ (/70)**	5.53 ± 6.02	15.68 ± 12.11	–	< 0.001^†^
**Baseline headache intensity**	1.59 ± 2.08	2.47 ± 2.24	–	0.012^†^
**Baseline neck pain intensity**	0.66 ± 1.31	2.99 ± 2.49	–	< 0.001^†^
**Baseline headache frequency**	0.97 ± 1.51	1.88 ± 1.78	–	0.005^†^
**Baseline neck pain frequency**	0.53 ± 1.37	3.22 ± 2.49	–	< 0.001^†^
**Home workstation** *Yes (%) / No (%)*	21.88 / 78.12	31.17 / 68.83	0.327	–
**Baseline headset wearing** *Yes (%) / No (%)*	56.25 / 43.75	66.23 / 33.77	0.325	–
*Follow-up variables*				
**Follow-up headset wearing** *Yes (%) / No (%)*	50.00 / 50.00	63.64 / 36.36	0.186	–
**Follow-up telecommuting hours**	5.39 ± 2.24	5.35 ± 1.85	–	0.921*
**Follow-up headset wearing hours**	1.43 ± 2.38	1.35 ± 1.98	–	0.401^†^

M = male, F = female, O = other gender (not identifying as either male of female), BMI = body mass index, HIT-6 = 6-item headache impact test, NBQ = Neck Bournemouth Questionnaire, sd = standard deviation, * = t-test, ^†^ = Wilcoxon test

Following the Spearman rank test analysis, only the NBQ was retained to represent baseline variables related to neck pain. The final stepwise multivariate regression model to determine associated factors of neck pain occurrence and the corresponding odds ratio are described in Table 6. In the end, 18.2% of the variance could be explained by the final model.

**Table 6:** Odds ratios, confidence intervals and *p*-values of factors associated with neck pain occurrence retained in the final stepwise model

Variables	Odds Ratio (95% CI)	P-value	Multivariate regression
**NBQ**	1.182 (1.102–1.269)	< 0.001	R^2^ = 0.182

NBQ = Neck Bournemouth Questionnaire, CI = Confidence Interval.

### Impact of headset on headache and neck pain intensity during the 5-day follow-up

For the second objective, t-test for independent groups showed no significant difference between participant that wore a headset during the follow-up and participants that did not wear a headset regarding participants headache mean intensity (*p* = 0.94) and participants neck pain mean intensity (*p* = 0.56).

## Discussion

Several studies have investigated psychological and physical risk factors of neck pain and headache in workers’ populations. Albeit the broad base of knowledge of these conditions and their respective risk factors, there are far less evidence available regarding these two conditions in the context of telecommuting. The COVID-19 pandemic has triggered an unprecedented and sudden increase in the proportion of workers that are now working from home and several experts and observers argue that such increase will partially persist over time [23–25]. Researchers have also raised concerns regarding this rapid shift to telecommuting and suggested that assessment of health risks and benefits of telecommuting are warranted [26]. This study sought to explore the potential physical risk factors of neck pain and headache in the telecommuters’ population.

### Headache

Our study showed that headache-related disability measured with the HIT-6 questionnaire was the only associated factors of headache occurrence following an adaptation to telecommuting. Only 9.4% of the variance could be explained by the final model that considered this associated factor. Although, it is well known that risk factors for headaches include several physical and psychological factors, these associated risk factors were only scarcely investigated in previous studies. A review investigating risk factors of chronic daily headache and migraine in Finnish municipal female employees highlighted several modifiable and non-modifiable risk factors [27]. Among the complex and multifactorial phenomenon associated with chronic daily headache and chronic migraine, the authors found significant association between daily headaches and sleep-related disorders, temporomandibular disorders, obesity, caffeine overuse, medication overuse, and high baseline headache frequency. Non-modifiable risk factors included old age, lower socioeconomic status, family history of chronic daily headache, significant recent life events (often considered as stressful events), and head injury. Other studies reported similar associated risk factors for chronic daily headaches in the general population, but also identified female gender, comorbid pain conditions as well as head and neck injury as potentially modifiable risk factors [28, 29]. Another study found that long working hours (more than 55 hrs per week) were associated with higher prevalence of headache. Our results suggest that most modifiable or non-modifiable risk factors in office workers such as female gender, working location (designed home workplace or not) and working hours were not present in telecommuters. According to these results, further ergonomic studies that involved measures of the workstation are needed to evaluate the long-term effects of telecommuting on headache.

### Neck pain

The results of our study showed that only neck pain-related disability was associated with future neck pain occurrence following a certain adaptation to telecommuting and 18.2% of the variance could be explained by the final model that considered this associated factor. Although one could assume that associated risk factors may be similar for office workers and telecommuters with similar employment conditions, the effects of telecommuting on physical health were only scarcely investigated. In their recent rapid review investigating the impact of telecommuting on individual workers’ mental and physical health, Oakman et al. (2020) only found three studies exploring physical health [4]. Their results suggest lower levels of pain (type or location not specified in the original study) in telecommuters and conflicting results with regard to self-reported health [5]. Given the lack of evidence concerning neck pain and headache associated risk factors in telecommuters, our results can only be compared with known risk factors in office workers. A recent review found strong evidence that individual, and work related physical risk factors for neck pain included gender (increased risk in female), previous history of neck complaints whereas only limited evidence or conflicting evidence for several other individual and work-related factors, including ergonomics [30]. Our results therefore suggest that, although telecommuting offers a flexible working context that is less limited by time and location, clinical neck pain risk factors in telecommuters are similar to those previously observed in office workers. However, our results did not show that gender was a neck pain associated factor. This result may be explained by the fact that most of our participants in both groups (without neck pain and with neck pain episodes) were females. These results echo previous work investigating the determinants of neck pain in the general working population [10].

Finally, contrary to our second hypothesis, the addition of a wearable device such as a headset during telecommuting did not have an impact on headache and/or neck pain in telecommuters. Even if the addition of a headset combined with a non-adaptative workstation could induce localized neck muscle fatigue, it has no impact on headache and neck pain intensity.

### Study strengths and limitations

The study was conducted remotely two months within the start of the COVID-19 pandemic in Canada and provides new insights into risk factors for headache and neck pain in telecommuters. It is, however, not without limitations. Attrition following the initial assessment was significant as several participants (32.7%) did not complete the 5-day follow-up. Systematic differences between responders and non-responders can introduce bias and lead to misleading interpretation of results. Such bias is believed to be limited in our study as statistical analyses showed that completers and non-completers were similar for baseline characteristics except for NBQ scores and baseline neck pain intensity. Even though the difference in disability between non-responders and responders was statistically and clinically significant [22], including non-responders could have only improved the prediction model. In fact, higher NBQ scores will increase the ability of this questionnaire to determine associated factors of future neck pain among telecommuters. Given the restrictions imposed by the COVID-19 pandemic, it was not possible to further assess the participants’ health complaints and therefore impossible to determine whether any associated and/or specific underlying condition may have been responsible for neck pain and headaches. Generalization to specific neck pain syndromes or headache types may be precarious because the present study did not discriminate headache and neck pain types. In addition, the small sample size may limit the strength of the conclusion that was made in this study. Reweighting based on population estimates was not possible as no study investigating telecommuting and its relation to headache and neck pain has been conducted previously. Thus, further research is required to confirm the present results among a larger sample size and to determine if these results can be generalized to the global population. Other potential modifiable and non-modifiable factors in office workers such as marital and family status, smoking, posture, break during work, high work load and mental stress should also be investigated [10, 31].

## Conclusion

The results of the present study showed that only headache-related disability score was an associated factor of headache occurrence and that only neck pain-related disability was an associated factor of neck pain occurrence following an acute adaptation to telecommuting. Wearing a headset was not associated with a higher neck pain and/or headache intensity. Future investigations are needed to confirm or infirm this tendency and to assess long term adaptations to telecommuting.

## Data Availability

The datasets used and/or analysed during the current study are available from the corresponding author on reasonable request.

## References

[1] Allen TD, Golden TD, Shockley KM (2015). How effective is telecommuting? Assessing the status of our scientific findings. Psychol Sci Public Interest.

[2] Aguilera A, Lethiais V, Rallet A, Proulhac L (2016). Home-based telework in France: characteristics, barriers and perspectives. Transp Res A Policy Pract.

[3] Tavares AI. Telework and health effects review. Int J Healthc. 2017;3(2):30. 10.5430/ijh.v3n2p30.

[4] Oakman J, Kinsman N, Stuckey R, Graham M, Weale V (2020). A rapid review of mental and physical health effects of working at home: how do we optimise health?. BMC Public Health.

[5] Giménez-Nadal JI, Molina JA, Velilla J (2019). Work time and well-being for workers at home: evidence from the American time use survey. Int J Manpow.

[6] Song Y, Gao J (2020). Does telework stress employees out? A study on working at home and subjective well-being for wage/salary workers. J Happiness Stud.

[7] National Research Centre for the Working Environment. From the study: Work environment and health in Denmark; 2016. https://arbejdsmiljoidanmark.nfa.dk/. Accessed 4 Jan 2021.

[8] Safiri S, Kolahi A-A, Hoy D, Buchbinder R, Mansournia MA, Bettampadi D (2020). Global, regional, and national burden of neck pain in the general population, 1990-2017: systematic analysis of the global burden of disease study 2017. BMJ..

[9] Stovner LJ, Nichols E, Steiner TJ, Abd-Allah F, Abdelalim A, Al-Raddadi RM (2018). Global, regional, and national burden of migraine and tension-type headache, 1990–2016: a systematic analysis for the global burden of disease study 2016. Lancet Neurol.

[10] Côté P, van der Velde G, Cassidy JD, Carroll LJ, Hogg-Johnson S, Holm LW, Carragee EJ, Haldeman S, Nordin M, Hurwitz EL, Guzman J, Peloso PM (2009). The burden and determinants of neck pain in workers: results of the bone and joint decade 2000-2010 task force on neck pain and its associated disorders. J Manip Physiol Ther.

[11] Sato K, Hayashino Y, Yamazaki S, Takegami M, Ono R, Otani K, Konno S, Kikuchi S, Fukuhara S (2012). Headache prevalence and long working hours: the role of physical inactivity. Public Health.

[12] Ye S, Jing Q, Wei C, Lu J. Risk factors of non-specific neck pain and low back pain in computer-using office workers in China: a cross-sectional study. BMJ Open. 2017;7(4):e014914. 10.1136/bmjopen-2016-014914. 10.1136/bmjopen-2016-014914 PMC5594207 28404613

[13] Vetvik KG, MacGregor EA (2017). Sex differences in the epidemiology, clinical features, and pathophysiology of migraine. Lancet Neurol.

[14] Jensen RH (2018). Tension-type headache - the Normal and Most prevalent headache. Headache..

[15] Fadinger H, Schymik J. The costs and benefits of home office during the covid-19 pandemic: evidence from infections and an input-output model for Germany. CEPR 2020;9:107–595 34.

[16] Lopez-Leon S, Forero DA, Ruiz-Díaz P. Recommendations for working from home during the COVID-19 pandemic (and beyond). Work. 2020;66(2):371–5. 10.3233/WOR-203187. 10.3233/WOR-203187 32568161

[17] Knight JF, Baber C (2007). Assessing the physical loading of wearable computers. Appl Ergon.

[18] Bigley GK. Headache. In: Walker HKHW, Hurst JW, editors. Clinical methods: the history, physical, and laboratory examinations. 3rd ed. Boston: Butterworths; 1990. Chapter 54. Available from: https://www.ncbi.nlm.nih.gov/books/NBK377/. Accessed 1 Feb 2021. 21250045

[19] Guzman J, Hurwitz EL, Carroll LJ, Haldeman S, Côté P, Carragee EJ, Peloso PM, van der Velde G, Holm LW, Hogg-Johnson S, Nordin M, Cassidy JD (2008). A new conceptual model of neck pain: linking onset, course, and care: the bone and joint decade 2000–2010 task force on neck pain and its associated disorders. Spine..

[20] Magnoux E, Freeman MA, Zlotnik G (2008). MIDAS and HIT-6 French translation: reliability and correlation between tests. Cephalalgia..

[21] Kosinski M, Bayliss M, Bjorner J, Ware J, Garber W, Batenhorst A (2003). A six-item short-form survey for measuring headache impact: the HIT-6™. Qual Life Res.

[22] Martel J, Dugas C, Lafond D, Descarreaux M (2009). Validation of the French version of the Bournemouth questionnaire. J Can Chiropr Assoc.

[23] Lyttelton T, Zang E, Musick K. Gender differences in telecommuting and implications for inequality at home and work. Available at SSRN 3645561. 2020.

[24] Conger, K. Facebook starts planning for permanent remote workers. The New York Times, B1; 2020. https://www.nytimes.com/2020/05/21/technology/facebook-remote-work-coronavirus.html. Accessed 4 Jan 2021.

[25] Contreras F, Baykal E, Abid G (2020). E-leadership and teleworking in times of COVID-19 and beyond: what we know and where do we go. Front Psychol.

[26] Bouziri H, Smith DR, Descatha A, Dab W, Jean K (2020). Working from home in the time of covid-19: how to best preserve occupational health?. Occup Environ Med.

[27] Malmberg-Ceder K, Vuorio T, Korhonen PE, Kautiainen H, Soinila S, Haanpää M (2020). The impact of self-reported recurrent headache on absenteeism and Presenteeism at work among Finnish municipal female employees. J Pain Res.

[28] Cho S-J, Chu MK (2015). Risk factors of chronic daily headache or chronic migraine. Curr Pain Headache Rep.

[29] Scher AI, Midgette LA, Lipton RB (2008). Risk factors for headache chronification. Headache.

[30] Kubo J, Goldstein BA, Cantley LF, Tessier-Sherman B, Galusha D, Slade MD, Chu IM, Cullen MR (2014). Contribution of health status and prevalent chronic disease to individual risk for workplace injury in the manufacturing environment. Occup Environ Med.

[31] Jensen R, Stovner LJ (2008). Epidemiology and comorbidity of headache. Lancet Neurol.

